# Linking crystal shape and dynamic undercooling: a new framework for inferring magmatic crystallization histories

**DOI:** 10.1007/s00410-025-02278-6

**Published:** 2025-12-02

**Authors:** Amanda Lindoo, Madeleine C. S. Humphreys, Charlotte Gordon, Martin F. Mangler, Edward W. Llewellin, Richard A. Brooker, Fabian B. Wadsworth, Eshbal Geifman

**Affiliations:** 1 Department of Earth Sciences, Durham University, Durham, DH1 3LE UK; 2 School of Earth Sciences, University of Bristol, Bristol, BS8 1RJ UK; 3 Department of Earth Sciences, University of Cambridge, Cambridge, CB2 3EQ UK

**Keywords:** Igneous textures, Undercooling, Crystallization, Crystal shape, Plagioclase

## Abstract

Magmas contain crystals exhibiting diverse shapes and sizes, yet the relationship between crystal shape (specifically aspect ratio) and undercooling ($\Delta T$), the driving force for crystallization, remains poorly constrained. Crystal shape should correlate with undercooling because undercooling governs the growth regime (interface-controlled versus diffusion-controlled) and thus the resulting crystal form. Prior experiments confirm that large nominal undercoolings drive transitions from polyhedral to hopper, skeletal, or dendritic forms. Large undercoolings reflect rapid decompression or cooling, differing from slower cooling rates typical of magmatic intrusions and storage systems. In such slowly cooled environments, crystals remain polyhedral, exhibiting subtle shape variations. Accurately quantifying crystal shape evolution at relatively low undercoolings could provide critical insights into crystallization histories, improving interpretations of the timescales and processes governing magma storage and eruption dynamics. Experimental verification of correlations between aspect ratios of polyhedral crystals and cooling rates remains inconclusive, possibly because nominal undercooling neglects the dynamic evolution of undercooling throughout crystallization. To address this, we introduce *average instantaneous undercooling* ($\overline{{\Delta T_{I} }}$), a metric capturing dynamic undercooling history during crystallization. Through controlled cooling experiments and numerical modelling, we demonstrate that higher $\overline{{\Delta T_{I} }}$ histories produce tabular, high aspect ratio plagioclase crystals, whereas lower $\overline{{\Delta T_{I} }}$ produces more prismatic crystals with lower aspect ratios. These variations in shape reflect undercooling-driven shifts in the predominant growth mechanism operating on different crystal faces. By quantitatively linking crystal shape to $\overline{{\Delta T_{I} }}$, our study provides a new approach for reconstructing crystallization histories in magmas under varying *pH2O-T-t* conditions.

**Supplementary Information:**

The online version contains supplementary material available at 10.1007/s00410-025-02278-6.

## Introduction

Constraining the crystallization histories of igneous rocks is fundamental for understanding magma transport, storage and differentiation in the Earth's crust, as well as the mechanisms and timescales driving magma mobilization and eruption. Experiments and field observations have demonstrated that cooling rate—or, more precisely, undercooling ($\Delta T$), a measure of the driving force for crystallization—strongly influences crystal size and morphology. Large undercoolings result in high nucleation rates, producing many, small crystals, whereas slow cooling promotes near-equilibrium conditions, resulting in fewer, larger crystals (Winkler 1949; Klein and Uhlman 1974; Gray 1978; Toramaru 1991; Cashman 1993). As a result, crystal size distributions can be used to estimate the timescales of cooling and crystallization given several important assumptions (e.g. Marsh 1988; Cashman and Marsh 1988; Higgins 2000). Volcanic dykes and sills have a planar geometry that allows cooling timescales to be estimated from thickness measurements of the intrusions. Crystal textures in such planar intrusions can then be indirectly related to cooling rates (Kirkpatrick 1976; Ikeda 1977; Cashman and Marsh 1988; Marsh 1988; Cashman 1993; Zieg and Marsh 2002; Holness 2014; 2017). However, inferring cooling timescales from textures alone, i.e. without independent constraints, requires assumptions about crystal growth rates, which are typically treated as isotropic (i.e. constant on all faces), size-independent, and constant over time, despite evidence to the contrary (Eberl et al. 2002; Arzilli et al. 2015; Befus and Andrews 2018; Andrews and Befus 2020; Mangler et al. 2023).

A common approach for linking crystal shape to thermal or decompression histories relies on nominal undercooling ($\Delta T_{N}$): the temperature difference between the initial saturation (liquidus) temperature of the crystallizing phase and the imposed sub-liquidus temperature where crystallisation is promoted. For decompression, the effective undercooling ($\Delta T_{Eff}$) is the temperature-equivalent shift in saturation caused by changes in pressure and melt volatile content. While $\Delta T_{N}$ provides a broad measure of the overall driving force for crystallization, it does not capture the evolving instantaneous undercooling during crystallization (Mollo and Hammer 2017). $\Delta T_{N}$ overlooks the continuous evolution of melt composition, shifting phase saturation conditions, and the dynamically changing nucleation and growth rates, potentially obscuring meaningful relationships between shape and undercooling.

**Fig. 1:**
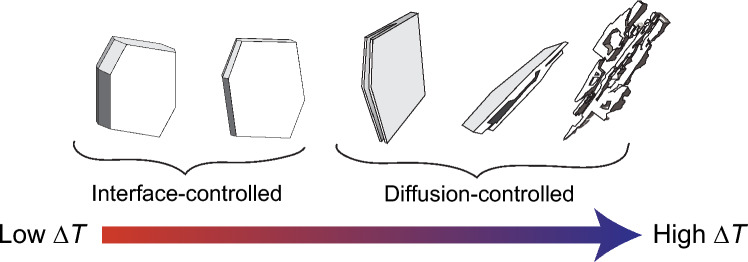
Classic morphological transition from polyhedral to skeletal shapes as growth shifts from interface- to diffusion-controlled growth regimes with increasing undercooling. Our study focuses specifically on interface-controlled shapes at low undercooling

Many studies have documented how plagioclase morphology evolves with $\Delta T_{N}$. At low $\Delta T_{N}$_,_ plagioclase exhibits polyhedral forms, whereas increasing $\Delta T_{N}$ favours hopper, skeletal, and dendritic morphologies (Fig. 1; Lofgren 1973, 1974, 1977, 1983; Donaldson et al. 1975; Kirkpatrick 1976; Fenn 1977; Swanson 1977; Nabelek et al. 1978; Walker et al. 1978; Kirkpatrick et al. 1979; Hammer and Rutherford 2002; Shea and Hammer 2013). These morphological changes reflect a shift from interface-controlled growth at low $\Delta T_{N}$, where growth is limited by the rate of atom attachment to the crystal interface, to diffusion-limited growth at high $\Delta T_{N}$, where growth is limited by the supply of components to and from the advancing crystal interface (Lasaga 1998). Moreover, an intermediate regime exists, where different crystal faces (or crystallographic directions) experience growth in different regimes—some remaining interface-controlled while others become diffusion-controlled, contributing to the anisotropic nature of plagioclase growth and the complexity of morphological transitions (Mangler et al. 2023).

**Fig. 2:**
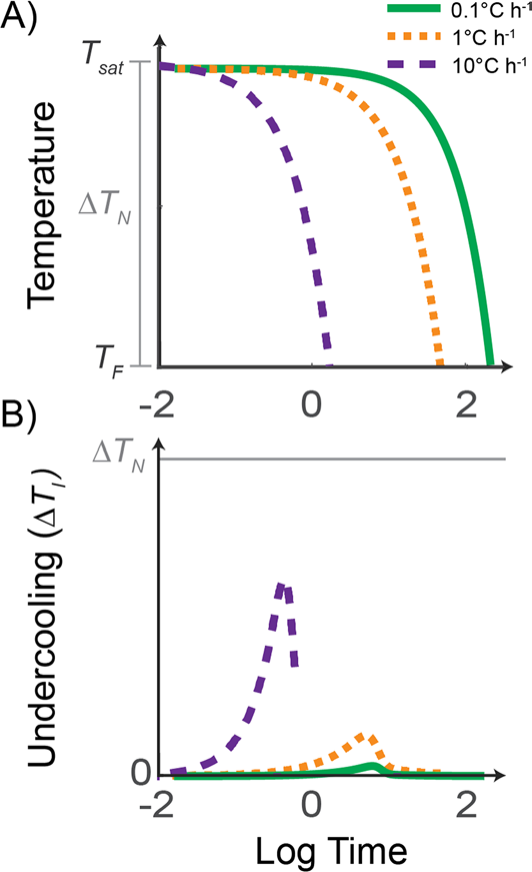
**A** Diagram illustrating three cooling experiments. The nominal undercooling (*ΔT*_*N*_) is commonly defined as the difference between the initial mineral saturation temperature ($T_{Sat}$) and the final temperature of the experiment ($T_{F}$). After an initial isothermal hold above the liquidus, experiments are cooled at different rates: $0.1^\circ {\text{C h}}^{ - 1}$ (green line), $1^\circ {\text{C h}}^{ - 1}$ (orange dashed line), and $10 ^\circ {\text{C h}}^{ - 1}$(purple dashed line). $\mathbf{B}\;\Delta T_{I}$ represents the real-time undercooling experienced during cooling. At faster cooling rates (purple and orange lines), crystallization may be delayed due to suppressed nucleation, such that $\Delta T_{I}$ initially increases during cooling, before decreasing exponentially as crystallization acts to reduce the Gibbs free energy. At relatively slower cooling rates (green line), this effect is diminished, and the overall average instantaneous undercooling ($\overline{{\Delta T_{I} }}$) is reduced

While the above studies have linked plagioclase morphology with overall changes in $\Delta T_{N}$, their approaches do not account for how undercooling evolves throughout crystallization. For example, at a given $\Delta T_{N}$, a slower cooling rate will result in a lower driving force for crystallization than a faster cooling rate and thus affect nucleation and growth kinetics (Fig. 2). To address this, we introduce the concept of average instantaneous undercooling ($\overline{{\Delta T_{I} }}$), which quantifies the time-averaged instantaneous undercooling ($\Delta T_{I}$) throughout the course of the experiment or crystallization interval. We conducted experiments at low to moderate $\Delta T_{N}$ (< 20 $^\circ {\text{C}}$), varying $\Delta T_{I}$ through controlled cooling rates ($0.09 - 720^\circ {\text{C h}}^{ - 1}$). By combining new and existing experimental data with a crystallization model that simulates $\Delta T_{I}$ over the experimental timescale, we calculated $\overline{{\Delta T_{I} }}$ for each experiment. Our results demonstrate that plagioclase shape correlates with $\overline{{\Delta T_{I} }}$ due to the anisotropic prevalence of different growth mechanisms operating on crystal faces (or crystallographic directions). This approach provides a new framework for interpreting crystal shapes formed during cooling or decompression, provided growth remains interface-controlled and unaffected by external factors such as crystal impingement.

## Background

Crystallization in magmas is governed by a complex interplay of thermodynamic and kinetic processes, which control the textures observed in igneous rocks. Understanding the balance of these processes is crucial for interpreting crystal textures. Crystallization, encompassing both nucleation and growth, acts to reduce the total Gibbs free energy of the system. The resulting crystal textures reflect the balance of rate-limiting factors, including the diffusion of elements through the melt and their attachment and migration to specific lattice sites at the crystal surface.

### Nucleation and growth: thermodynamic and kinetic controls

Crystallization begins with nucleation, where a stable nucleus with the properties of the macroscopic solid forms from the melt once the system surpasses a critical energy barrier, $\Delta G^{*}$. This energy barrier is a function of undercooling and can be expressed as (Spohn et al. 1988):

1 $$ \Delta G^{*} = \frac{{4s\sigma^{3} T_{Sat}^{2} }}{{\Delta H_{f}^{2} \left( {\Delta T} \right)^{2} }}, $$

here *s* is a shape factor, $\sigma$ is the interfacial energy at the crystal-melt interface [${\text{J}}\, {\text{m}}^{ - 2}$], $\Delta H_{f}$ is the enthalpy of fusion [${\text{J}}\, {\text{mol}}^{ - 1}$], $T_{Sat}$ is the phase saturation temperature [K], and $\Delta T$ is the undercooling, given by $\Delta T = T_{Sat} - T$, where $T$ is the temperature at a given time. This equation is a simplification for an isotropic case, where interfacial energies are assumed uniform across all crystal faces. However, for anisotropic minerals such as plagioclase, $\Delta G^{*}$ is assumed to vary by crystallographic face due to differences in interfacial energy. The nucleation rate ($I$) can be expressed as (Spohn et al. 1988):

2 $$ I\left( T \right) = I_{0} {\text{exp}}\left( { - \frac{{\Delta G_{D} }}{RT}} \right)\left[ {x + \left( {1 - x} \right){\text{exp}}\left( { - \frac{{\Delta G^{*} }}{RT}} \right)} \right], $$

Nucleation rate depends on the frequency of attachment attempts and number density of reactants in the melt $I_{0}$[${\text{m}}^{ - 3} {\text{ s}}^{ - 1}$], as well as the probability of overcoming $\Delta G^{*}$ and the activation energy for diffusion $\Delta G_{D}$[${\text{J}}\, {\text{mol}}^{ - 1}$]. Here, $R$ is the gas constant [${\text{J}}\, {\text{mol}}^{ - 1} {\text{K}}^{ - 1}$] and $x$ accounts for heterogeneous nucleation, incorporating the impact of impurities or pre-existing nuclei. Once nucleation has occurred, crystal growth proceeds at a rate determined by the instantaneous undercooling, reflecting a balance of interface and diffusion kinetics. Assuming interface-controlled growth, the growth rate ($U$) is expressed as (Spohn et al. 1988):

3 $$ U\left( T \right) = U_{0} \left[ {1 - exp\left( {\frac{{\Delta H_{f} \left( {\Delta T} \right)}}{{RTT_{Sat} }}} \right)} \right]exp\left( { - \frac{{\Delta G_{D} }}{RT}} \right), $$

Where $U_{0}$ is a constant dependent on the growth mechanism, atomic layer thickness, and the number of available sites on the crystal surface.

### Crystallization dynamics and how to describe them: nominal vs instantaneous undercooling

Temperature affects the melt structure by influencing the size, distribution and stability of atomic clusters that serve as potential nucleation sites. When the melt is heated well above its liquidus temperature, many of these clusters are destroyed, reducing the number available to initiate subsequent crystallisation (Donaldson 1979; Lofgren 1983; First et al. 2020). Upon cooling, the melt must reorganize to adjust to the new temperature, forming a new distribution of clusters suitable for nucleation. This reorganization takes time, known as the nucleation lag ($\tau$), or incubation time, which represents the period needed for nucleation to commence once the melt has reached a temperature at or below the saturation temperature. This lag can be especially pronounced after extensive superheating, as the highly disordered state of the melt inhibits nucleation (First et al. 2020). Previous studies have shown that nucleation delay is inversely proportional to undercooling, such that a smaller $\Delta T$, may delay or even prevent nucleation (Fenn 1977, Gibb 1974; Lofgren 1974; Swanson 1977; Nabelek et al. 1978; Donaldson 1979, Corrigan 1982; Rusiecka et al. 2020). Nucleation delay is described by (Fokin et al. 2006):

4 $$ \tau = \frac{{16k_{B} T\sigma }}{{3\Delta G_{V}^{2} \lambda^{2} D}} $$

here $k_{B}$ is the Boltzmann constant [${\text{J K}}^{ - 1}$]. Diffusivity, $D$, the rate of transport and arrival of atoms to the crystal surface can be estimated using the Stokes-Einstein relation (Ree and Eyring 1958; Donaldson 1975):

5 $$ D = \frac{{k_{B} T}}{\lambda \eta } $$

The melt viscosity $\eta$ [Pa s] can be estimated using viscosity models based on the melt composition (Giordano et al. 2008). The slowest diffusing elements, which act as rate-limiting factors, are represented by $\lambda$, the radius of the diffusing element ($\lambda$ = 0.26 nm for Si^4+^). Since diffusion rates decrease with cooling, growth initially accelerates to a maximum but slows as melt viscosity increases and diffusion becomes the limiting factor (Kirkpatrick et al. 1979; Lasaga 1998).

While nominal undercooling ($\Delta T_{N}$) is often used to characterise the driving force for crystallization, this simplification ignores dynamic processes such as phase saturation that evolve in response to crystallization (Mollo and Hammer 2017). To address this complexity, we define instantaneous undercooling ($\Delta T_{I}$) as the difference between the run temperature and the plagioclase saturation temperature for the melt in its current state (i.e., after any crystallization that has occurred). Because crystallization changes melt composition, the saturation temperature shifts and the instantaneous undercooling evolves with time. This history can be summarized as the average instantaneous undercooling ($\overline{{\Delta T_{I} }}$). Since $\Delta T_{I}$ directly controls nucleation and growth rates, $\overline{{\Delta T_{I} }}$ provides a better quantitative link between crystallization history and the resulting crystal textures (Lofgren 1980; Spohn 1988; Cashman 1993; Sunagawa 2009).

### Anisotropic crystal growth

The morphology of anisotropic minerals crystallizing at low $\Delta T_{I}$ is dictated by relative differences in growth rates of individual crystal faces, determined by their unique interfacial energies and related to crystal symmetry and bond strengths (Wulff 1901; Kundin et al. 2023). As $\Delta T_{I}$ increases, the dominant growth mechanism gradually shifts, influencing the relative growth rates of different faces and thus impacting crystal shape. At low $\Delta T_{I}$, growth is predominantly accommodated by screw dislocations. With increasing $\Delta T_{I}$, the surface nucleation of new layers (birth-and-spread growth) becomes increasingly more prevalent, eventually becoming dominant. At even higher $\Delta T_{I}$, continuous growth of atomically rough surfaces may occur, although this mechanism is thought to be uncommon in silicates (Jackson et al. 1967). Sunagawa (2009) proposed thresholds for changes in the dominant growth mechanism (Online Resource 1). However, these changes are likely not discrete but rather better described by a gradual shift in the relative importance of each mechanism with increasing undercooling, influenced by the complexity of the atomic structure. Consequently, multiple growth mechanisms or differing growth rates may operate simultaneously on adjacent crystal faces, even under identical $\Delta T_{I}$. Crystal shapes, therefore, can vary considerably with undercooling and growth history, even within the broader regime of interface-controlled growth.

### Scope of prior work and the need for $\overline{{\Delta T_{I} }}$

Previous experimental studies on plagioclase morphology have explored a wide range of nominal undercoolings (typically $\Delta T_{N} \approx 25 - 125^\circ C$) under moderate to rapid thermal or decompression pathways (step-changes in temperature or pressure). These studies clearly highlighted the distinct transition from interface- and diffusion-controlled growth regimes and the associated shift toward hopper, skeletal, and dendritic morphologies (Fig. 1; Lofgren 1973, 1974, 1977, 1983; Gibb 1974; Donaldson et al. 1975; Kirkpatrick 1976; Fenn 1977; Swanson 1977; Nabelek et al. 1978; Walker et al. 1978; Kirkpatrick et al. 1979; Hammer and Rutherford 2002; Couch et al. 2003; Conte et al. 2006; Brugger and Hammer 2010; Shea and Hammer 2013; Arzilli et al. 2015; Befus and Andrews 2018). In contrast, lower $\Delta T_{N} { }$ (e.g., < 30 °C) and slower crystallization rates relevant to subtle changes in crystal aspect ratios and conditions expected of lava flow interiors and magmatic intrusions, have been less explored.

A few studies have provided some insight into this area. Billon et al. (2025) employed slower cooling rates ($1$,$3, 9^\circ {\text{C h}}^{ - 1}$) and observed a general increase in plagioclase aspect ratio with increasing cooling rate. Mangler et al. (2023) found that at low $\Delta T_{N}$ (5–30 °C), plagioclase crystals evolve from prismatic shapes (low aspect ratio) to bladed forms (higher aspect ratio) as crystals grow, eventually stabilizing at a steady-state beyond volumes of ~100 µm^3^. However, these studies do not provide a quantitative link between undercooling and the mature shape of polyhedral crystals under slowly cooled conditions. Thus, we introduce $\overline{{\Delta T_{I} }}$, which integrates $\Delta T_{I} \left( t \right)$ over the crystallization interval and links time-varying undercooling histories to observed crystal aspect ratios and can thereby offer insights into the evolution of magmatic systems. The term $\Delta T_{I}$ is closely related to the supersaturation term ($\Delta \varphi$) of Befus and Andrews (2018), expressed in units of crystallinity; both quantify the system’s departure from equilibrium along the cooling/decompression history.

## Methods

### High temperature experiments

To observe how the shape of plagioclase evolves over time under low to moderate ($\Delta T_{N} =$ 12–20 °C) undercoolings, we performed a set of cooling experiments in a one atmosphere (1-atm) furnace to ensure accurate temperature control. The experiments were carried out under anhydrous conditions with synthetic, high-alumina basalt starting composition (${\text{SiO}}_{2} :49.84\left( {0.18} \right)$; ${\text{Al}}_{2} {\text{O}}_{3} :{ }19.60\left( {0.03} \right)$, ${\text{Na}}_{2} {\text{O}}:{ }3.24\left( {0.07} \right)$, ${\text{MgO}}:4.92\left( {0.07} \right)$, ${\text{ FeO}}:{ }9.52\left( {0.07} \right)$, ${\text{ CaO}}:{ }9.46\left( {0.02} \right)$ ,${\text{ K}}_{2} {\text{O}}:{ }0.77\left( {0.01} \right)$, ${\text{ TiO}}_{2} :{ }1.26{ }\left( {0.01} \right)$) in which plagioclase is the sole phase from *T*~1275 °C until *T*~1170 °C (Baker and Eggler 1983).

The starting material was prepared by drying reagent-grade oxides and carbonates at temperatures of $1000$ and 500 °C, respectively, for $12 - 24$ hours. The powders were then combined to form a composition similar to the high-Al basalt utilised by Baker and Eggler (AT-1; 1983). The mixture was ground in ethanol, slowly heated from 500 to 1000 ºC over five hours, and then held at 1000 °C overnight to decarbonate. Following decarbonation, FeO and ${\text{Fe}}_{2} {\text{O}}_{3}$ powders were added to the mixture and ground for 45 minutes under ethanol using a mortar and pestle. The fine powder was loaded into a Pt crucible and lowered into a gas-buffered vertical furnace maintained at NNO + 0.8. After an hour at 1300 °C, the material was quenched to create glass and subsequently reground and fused again to increase compositional homogeneity. Finally, the glass was powdered a third time and used to form beads for 1-atm experiments. Similar experiments were also performed using small glassy shards of a Blue Glassy Pahoehoe sample, previously used by Mangler et al. (2023).

One-atmosphere (1-atm) experiments were performed at the University of Bristol using a GERO vertical tube furnace equipped with a CO–CO_2_ gas-mixing system and drop-quench capability. To prepare the samples, we formed beads on Pt loops, approximately 2 mm in diameter by binding the sample powder with polyvinyl alcohol. The Pt loop was then suspended across the top of a Pt crucible, which was held in a custom grip that enables a drop quench. Basaltic compositions are susceptible to Fe-loss during glass fusion and during experiments due to interactions between the Pt crucible used for synthesizing the starting material and the Pt-wire supporting 1-atm beads. While Fe-loss did occur, it is not expected to impact our study because plagioclase remains the liquidus phase across a range of oxygen fugacities, and our focus is exclusively on plagioclase growth, which is unaffected by variations in Fe content in the melt across the temperatures of interest (Baker and Eggler 1983).

Temperature measurements were obtained through a type-B (Pt_70_Rh_30_/PtRh_6_) thermocouple positioned less than 5 mm above the sample bead. The thermal gradient within the Pt crucible was found to be negligible, with a variation of only $\pm 1$ °C within $15$ mm below the primary thermocouple. The samples were annealed at 1280 °C, 5 °C above the liquidus, for a minimum of two hours. The goal of the supraliquidus step was to ensure the melting of any crystals that nucleated on the glass powder surfaces during the heating process. One experiment was quenched $15$ minutes after being brought to 1280 °C to examine crystal formation during heating. We found that a $2$-h hold period at 1280 °C was required to minimise the presence of these crystals. Additionally, we found that annealing at temperatures exceeding 5 °C above the liquidus temperature disrupted the establishment of an interface-controlled growth regime, as trial experiments held at temperatures >10 °C above the liquidus exhibited hopper crystal shapes after cooling, indicative of diffusion-controlled growth. This finding aligns with prior research, suggesting a correlation between superheating magnitude and crystal morphology (Nabelek et al. 1978; Donaldson 1979; Corrigan 1982; First et al. 2020). After the supraliquidus step, we applied continuous cooling ramps of $0.09, 0.8, 1.6, 24$, and 60 °C h^−1^ to bring the samples to final temperatures of $1262$ or 1255 °C (Table 1; Online Resource 1). Continuous ramps effectively function as a smooth progression of infinitely small, incremental changes and therefore, are more accurately characterized by the cooling rate rather than the nominal undercooling (Mollo and Hammer 2017). These incremental steps introduce smaller changes in $\Delta G$ during cooling, leading to the development of crystal textures that differ from those produced by single-step cooling (Brugger and Hammer 2010). Considering that continuous cooling best approximates the cooling of magmatic intrusions, we employed mostly continuous cooling ramps. One experiment (1atm-3) was held for $12$ h following its cooling ramp. Another was cooled using the single-step method for reference (1atm-4, Table 1). At the conclusion of each experiment, the sample was released from its ceramic holder, dropping into a water bath underneath the furnace, quenching the melt to a glass within ~$2$ seconds.We confirmed the temperature of the 1-atm plagioclase liquidus by performing isothermal experiments $\pm 10$ °C from 1275 °C; the liquidus observed by Baker and Eggler (1983) at $1$-atm.

**Table 1:** Run conditions

Run no.	Type	Pre-experiment anneal (°C)	Cooling rate (°C h^−1^)	*T*_*Final*_ (°C)	$\Delta T_{N} { }$ (°C)	Run duration (h)^£^	$Max \Delta T_{I}$* (°C)	$\overline{{\Delta T_{I} }}$** (°C)
1atm_1	Ramp	2h at 1280	0.8	1262	13	16.3	9.0	5.7
1atm_2	Ramp	12h at 1280	1.6	1262	13	8.1	4.4	0.9
1atm_3^*^	Ramp	2h at 1280	1.6	1262	13	20.1	12.9	8.4
1atm_8	Ramp	2h at 1280	60	1255	20	0.33	20.0	10.0
1atm_9	Ramp	2h at 1280	24	1255	20	0.83	14.6	9.1
1atm_BGP26	Ramp	2h at 1180	24	1155	20	0.83	20.0	10.0
1atm_BGP27	Ramp	3h at 1180	1	1155	20	20	18.7	9.7
1atm_BGP28	Ramp	0.8h at 1180	24	1155	20	0.83	19.7	9.4
1atm_BGP29	Ramp	2h at 1180	0.09	1155	20	222.22	15.5	9.2
1atm_4	SSC^†^	2h at 1280	720	1262	13	0.02	13.0	1.5

*Maximum value of instantaneous undercooling ($\Delta T_{I}$)

**Time-averaged instantaneous undercooling

^†^Single-step cooling

^£^Does not include pre-experiment anneal time

### Crystal texture analyses

Beads from 1-atm experiments were embedded in epoxy, allowed to cure overnight, and ground or cut in half to expose a cross-section. The surface was further ground using SiC grit papers and polished with a series of diamond pastes down to 0.3 µm. The polished sections were then carbon coated for electron microscopy.

We collected backscatter electron (BSE) images of the experiments using a Hitachi S-3500N scanning electron microscope (SEM) at the University of Bristol, operating at an accelerating voltage of $15$ kV. To minimize errors in volume fraction estimates caused by variations in crystal texture (e.g., melt-dominated regions), complete panoramas of the sample surfaces were obtained at Durham University using a Hitachi SU-70 FEG SEM. Plagioclase crystals not touching the edge of the images were manually outlined, and their number ($n$), 2D areas (*A*), lengths (*l*), and widths (*w*) were measured using the best-fit ellipse output in *ImageJ*. Plagioclase crystallinity ($\phi$) was calculated by summing the areas of plagioclase crystals and dividing by the total area analyzed from panoramas ($A_{Total}$). This allowed the calculation of areal number density ($N_{A} = n/A_{Total}$; mm^-2^) and volumetric number density $\left( {N_{V} = N_{A} /\sqrt {A_{plag} /n_{plag} } } \right)$ (DeHoff and Rhines 1968; Hammer et al. 1999). These values were used to calculate the characteristic (average) crystal size $\left( {S_{n} = \sqrt {\phi /N_{A} } } \right)$ and average crystal volume ($V_{avg} = \phi /N_{V} ;$Table 4).

Deriving 3D crystal shapes has been a long-standing challenge in textural studies. Plagioclase crystal sections are often manually outlined from backscattered electron (BSE) images due to their similarity in grayscale values to the surrounding glass matrix. Generating a statistically robust characterisation of the 2D width-to-length (*w/l*) distribution requires many crystal section measurements, which can be challenging for experimental studies with low crystal number densities. The 2D distribution is then compared to numerical models that simulate random sectioning of cuboids with known short: intermediate: long (*S:I:L*) dimensions to produce representative *w/l* distributions (e.g., *CSDCorrections*, *CSDSlice,* and *ShapeCalc*) (Higgins 2000; Morgan and Jerram 2006; Mangler et al. 2022). These models remain the most reliable tools for reconstructing 3D shapes from 2D data, although inherent uncertainties in determining the longest dimension (*L*) remain, as the likelihood of sectioning the most elongate axis fully within a cross-section is low. Zingg diagrams, which plot the ratio of *S/I* against *I/L,* enable visualization of 3D morphologies, classifying shapes into quadrants that represent equant (*S ≈ I ≈L*), prismatic (*S ≈ I < L*), tabular (*S < I ≈ L*), and bladed (*S < I < L*) shapes (Zingg 1935; Mangler et al. 2022). For this study, 3D crystal habits were estimated by importing 2D intersection length and width measurements into *ShapeCalc* (Mangler et al. 2022). We treated each experiment as comprising a single shape population.

### Modelling crystallization

To estimate the evolution of the time-averaged instantaneous undercooling $\overline{{\Delta T_{I} }}$ [K] during our experiments, we developed a MATLAB-based numerical model that simulates plagioclase crystallization under various cooling rates. This model employs a nucleation-growth framework following Spohn et al. (1988) and is similar to the *SNGPlag* program (Andrews and Befus 2020), incorporating time-dependent undercooling and melt compositional evolution. However, we additionally include the possibility of nucleation delays, and the primary outputs are the time-averaged and maximum instantaneous undercoolings. The publication of our source code allows for further adaption to simulate crystal size distributions and incorporation of anisotropic growth.

Equilibrium crystallinity of plagioclase, $\phi_{Eq}$ [−], and the corresponding Gibbs free energy of formation ($\Delta G$, J mol⁻^1^) were calculated as functions of temperature using *MELTS* (Ghiorso and Sack 1995; Asimov and Ghiorso 1998) for two melt compositions (BEAT1-2 and BGP). The tabulated $T - \phi_{Eq}$ and $T - \Delta G$ data were each converted into continuously differentiable functions via piecewise-cubic Hermite interpolation (“pchip”), preserving the data trend and avoiding overshoot. These interpolants enabled continuous tracking of feldspar saturation during cooling. In parallel, melt viscosity, $\eta$ [Pa s] was computed at each crystallinity step using the Giordano et al. (2008) VFT model. All interpolants and conditional evaluations were implemented in MATLAB scripts.

For each experiment, a temperature-time profile was constructed based on nominal undercooling $\Delta T_{N}$ [K], imposed cooling rate, and any isothermal hold periods. Thus, the model advances iteratively through discrete time steps $\Delta t$ [s], performing the following computations at each interval:

[1] The model begins by finding the saturation temperature $T_{Sat}$ [K] at that start of crystallization (the liquidus temperature, $\phi = 0$). The first timestep defines the instantaneous undercooling $\Delta T_{I}$[K] as:

6 $$ \Delta T_{I,1} = T_{Sat,1} - T_{1} , $$

At each subsequent timestep, we update the crystallized fraction $$\phi$$ and recompute the corresponding saturation temperature $$ T_{sat}$$ using the established $$\phi_{Eq} \left( T \right)$$ relationships, reflecting whether equilibrium crystallinity is maintained. This procedure ensures that $$\Delta T_{I}$$ reflects the response of the system. Instantaneous undercooling is then:

7 $$ \Delta T_{I,k} = T_{Sat,k} - T_{k} $$

[2] To account for the time delay preceding nucleation, we implemented Eq. (4). After surpassing the nucleation delay ($\tau \left[ s \right]$), the number density of crystals $n$ [# m^−3^] at each timestep is updated:

8 $$ n_{k} = n_{k - 1} + I_{k} \Delta t, $$

where the subscript $k$ is the timestep index. The nucleation rate $I_{k}$ [m^-3^s^-1^] for the timestep is calculated using the expressions from Spohn et al. (1988; see Eqs. 1–2) and established nucleation parameters listed in Table 2.

**Table 2:** Parameters used in nucleation and growth equations

Parameter	Value	References
$\sigma$; Bulk surface free energy (${\text{J m}}^{ - 2}$)	1.2e−1	Fokin et al. (2006), Mollard et al. (2020)
$\Delta H_{f}$; Enthalpy of fusion (${\text{J mol}}^{ - 1}$)	7.43e+4	Muncill and Lasaga (1988), Hammer (2004)
$\Delta G_{D}$; Activation energy for diffusion (${\text{J mol}}^{ - 1}$)	2.88e+5	Taniguchi (1992), Toplis and Dingwell (2004)
$s$; shape factor	5.00e+2	Mollard et al. (2020)
$x$; fraction of pre-existing nucleation sites	1e−3	
$\lambda$; radius of slowest diffusing element (m)	2.6e−11	Shannon (1976), Baker et al. (2020)
Pre-exponential factors:	$I_{0} \left( {{\text{m}}^{ - 3} {\text{ s}}^{ - 1} } \right)$	$U_{0}$ (${\text{m s}}^{ - 1}$)
1atm-1	2.65e+19	− 5.14e+1
1atm-2	2.33e+19	− 8.85e+3
1atm-3	7.34e+17	− 9.72e+1
1atm-8	1.62e+18	− 1.35e+2
1atm-9	6.26e+20	− 8.41e+2
1atm-BGP26	2.54e+20	− 8.29e+2
1atm-BGP27	7.83e+17	− 1.59e+2
1atm-BGP28	5.09e+21	− 7.99e+2
1atm-BGP29	9.34e+16	− 1.86e+1
1atm-4	5.85e+19	− 2.38e+2

[3] To calibrate our crystallization model, we systematically varied two key input parameters: the pre-exponential factors, $I_{0}$ [${\text{m}}^{{ - 3{ }}}$ ${\text{s}}^{ - 1}$] and $U_{0}$ [m ${\text{s}}^{ - 1}$], used in the nucleation and growth equations (Eqs. 2 and 3). Theoretical estimates of $I_{0}$ can be extremely large (on the order of $10^{43}$), greatly exceeding experimental observations (e.g., $\sim 10^{21}$; Toramaru and Kichise 2023). To determine the best coefficients for each of our experiments, we performed a coarse, random search over logarithmically spaced ranges of $I_{0}$ ($10^{15}$ to $10^{25}$) and $U_{0}$ ($- 10^{1}$ to $- 10^{6}$) to identify parameter pairs whose simulated crystal number densities ($N_{V} [{\text{m}}^{{ - 3{ }}}]$) and volume fraction ($\phi$) lie close to the experimentally measured values. For each experimental cooling pathway, we ran our crystallization model using each candidate pair, calculating modelled $N_{V}$ and $\phi$. We assessed the candidate pairs by computing a combined mismatch metric between simulated and experimental crystal number densities and crystallinities using the following:

9 $$ N_{{V_{Mismatch} }} = \left( {log10\left( {N_{{V_{End} }} } \right) - log10\left( {N_{{V_{Target} }} } \right)} \right)/log10\left( {N_{{V_{Target} }} } \right) , $$

10 $$ \phi_{Mismatch} = \left( {\phi_{End} - \phi_{Target} } \right)/\phi_{Target} , $$

11 $$ Error = \sqrt {\left( {N_{{V_{Mismatch} }}^{2} } \right) + \left( {\phi_{Mismatch}^{2} } \right)} , $$

The best-guess $I_{0} /U_{0}$ combinations were used as initial guesses for a local refinement using MATLAB’s MultiStart (global optimization toolbox). We defined a tolerance of 0.2 log units for the mismatch between simulated and actual $N_{V}$, and a tolerance of 0.01 for $\phi$*.* The parameter combination yielding the lowest error value was then selected as the calibrated $I_{0}$ and $U_{0}$. For experiments that had crystal fractions exceeding those predicted by MELTS, we used the MELTS equilibrium $\phi$ for optimization. Therefore, these experiments have inherently higher errors associated with their modelled undercooling history, generally producing lower undercoolings at a given time. This calibration procedure must be conducted using the crystal number densities and volume fractions from the sample of interest, otherwise the resulting undercooling history will not be representative.

With each experiment’s calibrated $I_{0} /U_{0}$ parameters, we then reran the crystallization model to calculate two key metrics: (1) the maximum instantaneous undercooling encountered during cooling and (2) the time-averaged undercooling over the entire experiment. Because long isothermal holds following a temperature step can obscure much of the undercooling signal, the maximum undercooling is particularly informative for experimental samples that underwent stepped cooling pathways (single-step or multi-step), whereas the time-averaged value better captures the overall degree of disequilibrium for continuously cooled samples, or natural samples.

[4] Following the determination of the nucleation rate and crystal number density for the timestep, the size of previously formed crystals is updated:

12 $$ r_{k} = r_{k - 1} + U_{k} \Delta t, $$

where r [m] is the current crystal radius. Crystals nucleated at timestep *k* are initialized at r = $U_{k} \Delta t$. Because nucleation spans multiple timesteps and the growth rate $U$ varies with the evolving $\Delta T_{I} \left( {\text{t}} \right)$, crystals experience different growth durations. Therefore, the model produces a polydisperse size distribution: earlier-nucleated cohorts are larger than later cohorts, and size contrasts are amplified as $U$ changes through time. Growth rates are calculated from Eq. (3).

[5] At each timestep, the volume fraction of crystals ($\phi$) is calculated under the assumption of spherical growth:

13 $$ \phi_{k} = \phi_{k - 1} + n_{k} \Delta \frac{4}{3}\pi r^{3} , $$

which adds the volume of newly nucleated crystals and the growth increment of previously existing cohorts. We modelled crystals as spheres for simplicity. Anisotropic shapes can be accommodated; however, this substitution does not change the evolution of $\overline{{\Delta T_{I} }}$.

[6] Finally, the maximum $\Delta T_{I}$ value is found and the $\Delta T_{I}$ values are integrated over the experiment and normalised to the total experimental duration ($t_{final}$) to calculate the time-averaged instantaneous undercooling $\overline{{\Delta T_{I} }}$:

14 $$ \overline{{\Delta T_{I} }} = \frac{1}{{t_{final} }}\mathop \smallint \limits_{t = 0}^{{t_{final} }} \Delta T_{I} dt $$

**Fig. 3:**
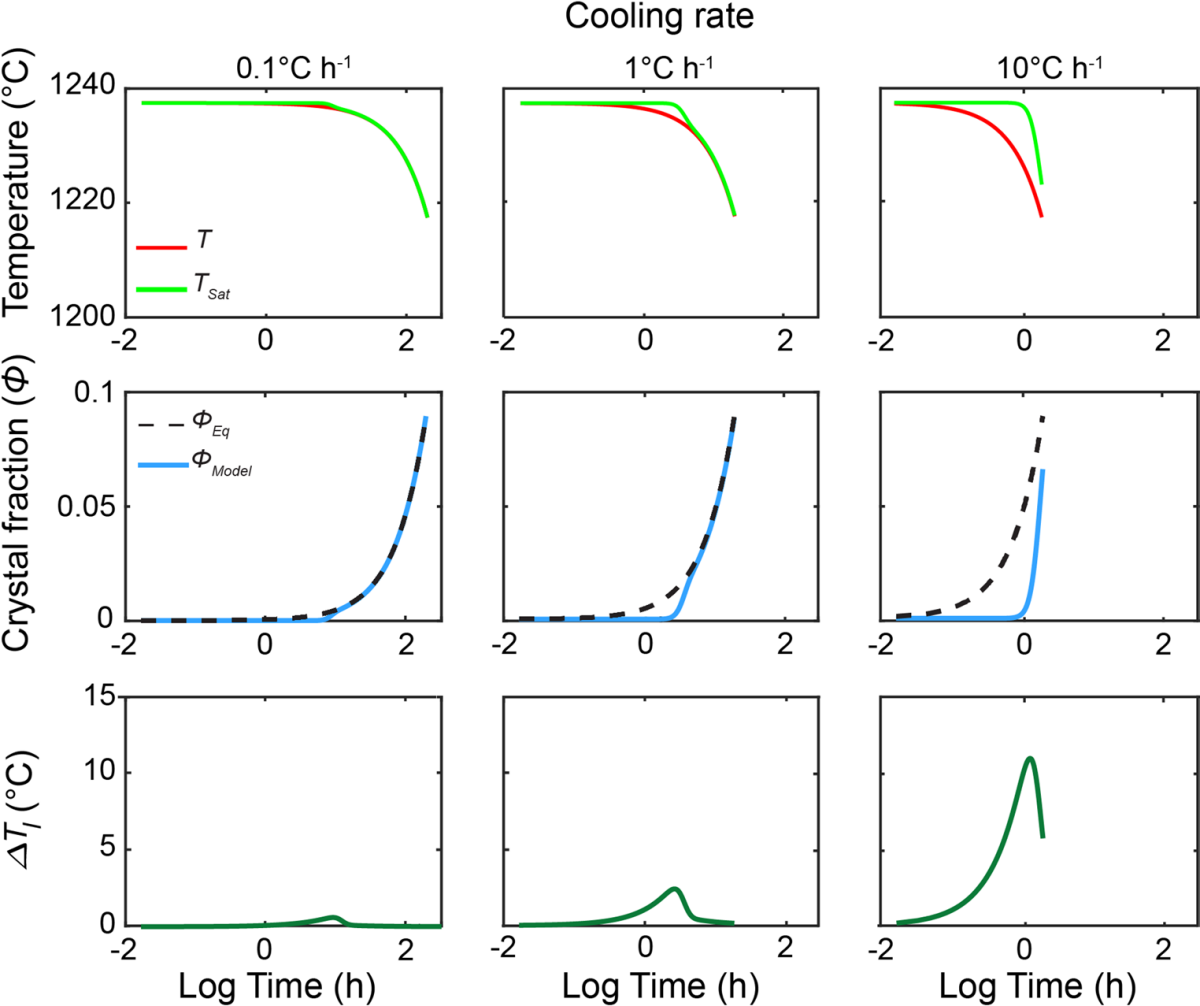
Outputs from three cooling rate simulations generated by the crystallization model. (Top row) Temperature-time profiles of the applied cooling rates (red line) and the evolution of the plagioclase saturation temperature, calculated from our model ($$T_{Sat} ; $$ green line). (Middle row) Comparison of equilibrium crystallinity predicted by MELTS ($$\Phi_{Eq}$$; black dashed line) with the crystallinity calculated from our model as a function of time ($$\Phi_{Model}$$; blue line). (Bottom row) Evolution of $$\Delta {T}_{I}$$ throughout the simulation (dark green line). These plots were produced using the parameters in Table 2 as well as pre-exponential factors of $${\text{I}}_{0} = 1{\text{e}} + 19{\text{ m}}^{ - 3} {\text{ s}}^{ - 1}$$ and $${\text{U}}_{0} = - 1{\text{e}} + 3$$ $${\text{m s}}^{ - 1}$$

The resulting maximum and average instantaneous undercooling provide quantitative measures of the degree of disequilibrium experienced during the crystallization timescale, providing a robust parameter for comparing the driving force for crystallization across different thermal (or *pH2O*) histories. Example outputs from the model, including the computed evolution of $\phi$ (blue lines) and $\Delta T_{I}$ (dark green lines) are illustrated in Fig. 3.

### EPMA analytical methods

The major element composition of the starting glass was analyzed using electron probe microanalysis (EPMA) on a JEOL JXA8530F FEG-EPMA at the School of Earth Sciences, University of Bristol. The analyses were conducted with an accelerating voltage of 15 kV and a beam current of 10 nA. A defocused beam with a 10 $\mu$m diameter was used for glass analyses, while a focused 2 µm beam was employed for plagioclase analyses. Calibration was performed using the following standards: albite (Si), sanidine (Al, K), apatite or wollastonite (Ca), NaCl (Na), St John’s Island Olivine (Mg), fayalite or hematite (Fe), TiO_2_ (Ti), and SrTiO_3_ (Sr). To prevent migration effects in glass, sodium and potassium were analyzed first. Working secondary standards for glass analyses included Columbia River basalt (BCR-2G) and Juan de Fuca basaltic glass (VG-2), while San Pedro Hills (SPH) Labradorite was used as the secondary standard for plagioclase analyses.

### EBSD methods

The relevant crystal faces and relative growth along *a*, *b*, and *c* directions were determined through Electron Back-Scatter Diffraction (EBSD) methods. EBSD maps were acquired using a FEI Quanta 650 SEM equipped with an Oxford Instruments Symmetry S3 detector and *AZtec* 6.1 acquisition software, in the Department of Earth Sciences, University of Cambridge. Prior to EBSD analysis, all samples were additionally hand-polished for ~$20$ minutes with colloidal silica. EBSD was performed using a $70^\circ$ specimen tilt under low vacuum, with no carbon coat. We used a $100$ *µ*m aperture, spot size of 4.5, and an accelerating voltage of $25$ kV, with typical working distances of ~20 mm. Patterns were collected using Speed 1 mode, with frame averaging of $1 - 3$, and indexed using the Optimised BD setting. Plagioclase was indexed against an anorthite match unit (American Mineralogist Crystal Structure Database no. $0000370$ from Foit and Peacor 1973). Maps were created with step sizes ranging from $0.25$ to $0.7 \mu {\text{m}}$. Fore-scatter diodes were used to capture images of the samples immediately prior to EBSD analysis, and EDS maps were collected simultaneously to confirm the phases and textures present.

**Fig. 4:**
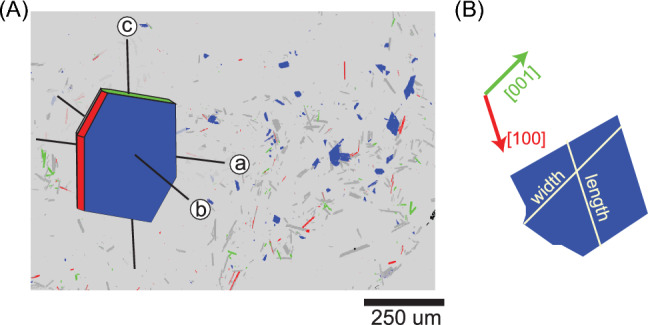
**A** Example crystallographic orientation map produced using the MTEX toolbox in MATLAB. Grains are coloured by the crystallographic direction perpendicular to the map plane: red for (100)*, blue for (010)* and green for (001)*. A representative annotated plagioclase crystal shape is overlaid for reference. **B** Example crystal viewed along the (010)* direction, with [100] and [001] axes indicated by arrows, informing the width and length measurements used

We de-noised EBSD data in *AZtecCrystal*, using a function that replaces wild spikes to remove mis-indexed single pixels. No filling or smoothing was applied. All further data processing and plotting were conducted using *MTEX* version 5.11.2, an open-source *MATLAB* toolbox (Bachmann et al. 2011). Grains with (100)*, (010)* and (001)* crystallographic directions within 22º of perpendicular to the plane of the EBSD map plane were identified, since such sections have two crystallographic axes lying approximately within the plane of the map. The lengths of the crystals along these axes were then measured (Fig. 4). For each sample, the dimensions of the ten longest crystals were averaged, and the standard deviation was calculated (Fenn 1977; Swanson 1977; Hammer and Rutherford 2002). These measurements were then used to determine the short: intermediate: long (*S:I:L*) dimensions of the crystal population, from which the *S/I* and *I/L* ratios were derived. To account for the variability in the crystal lengths, the standard deviations of each axis were incorporated into error propagation calculations for the EBSD-derived *S/I* and *I/L* ratios.

For a subset of six samples, we compared the *S/I* and *I/L* values derived from EBSD and *ShapeCalc* to evaluate the reliability of *ShapeCalc* as a method for reconstructing 3D crystal shapes (Table 4; Online Resource 1). *ShapeCalc*-derived *S/I* values tended to be higher than those derived from EBSD measurements. This discrepancy is expected, as the two approaches measure fundamentally different parameters: geometric axes (*ShapeCalc*) versus crystallographic axes lengths (EBSD). Uncertainties were consistently high for *I/L* ratios derived from both *ShapeCalc* and EBSD data, limiting their utility. Therefore, our primary discussion focuses on *S/I* values, and we rely on EBSD-derived measurements when differentiating subtle variations in shape.

## Results

Our experiments were conducted to extend the range of experimental conditions available from previous studies. Below, we present our observations alongside a previous experimental study on a mafic system that included comprehensive crystal length and width data (Mangler et al. 2023). That study employed experimental conditions that closely aligned with ours but varied in cooling rates, nominal undercoolings ($\Delta T_{N}$), and melt diffusivities. Mangler et al. (2023) performed experiments at low nominal undercoolings ($\Delta T_{N}$ < 20°C) but generally faster cooling rates ($10 - 258^\circ {\text{C h}}^{ - 1}$) using the BGP basalt composition. Additionally, minimal superheating was applied before cooling ($5^\circ C$ above the liquidus). We used the *S/I* values reported by Mangler et al. (2023; stars in Figs. 6, 8, 9 and 10), given the similarity in our methodology to calculate 3D shapes.

**Fig. 5:**
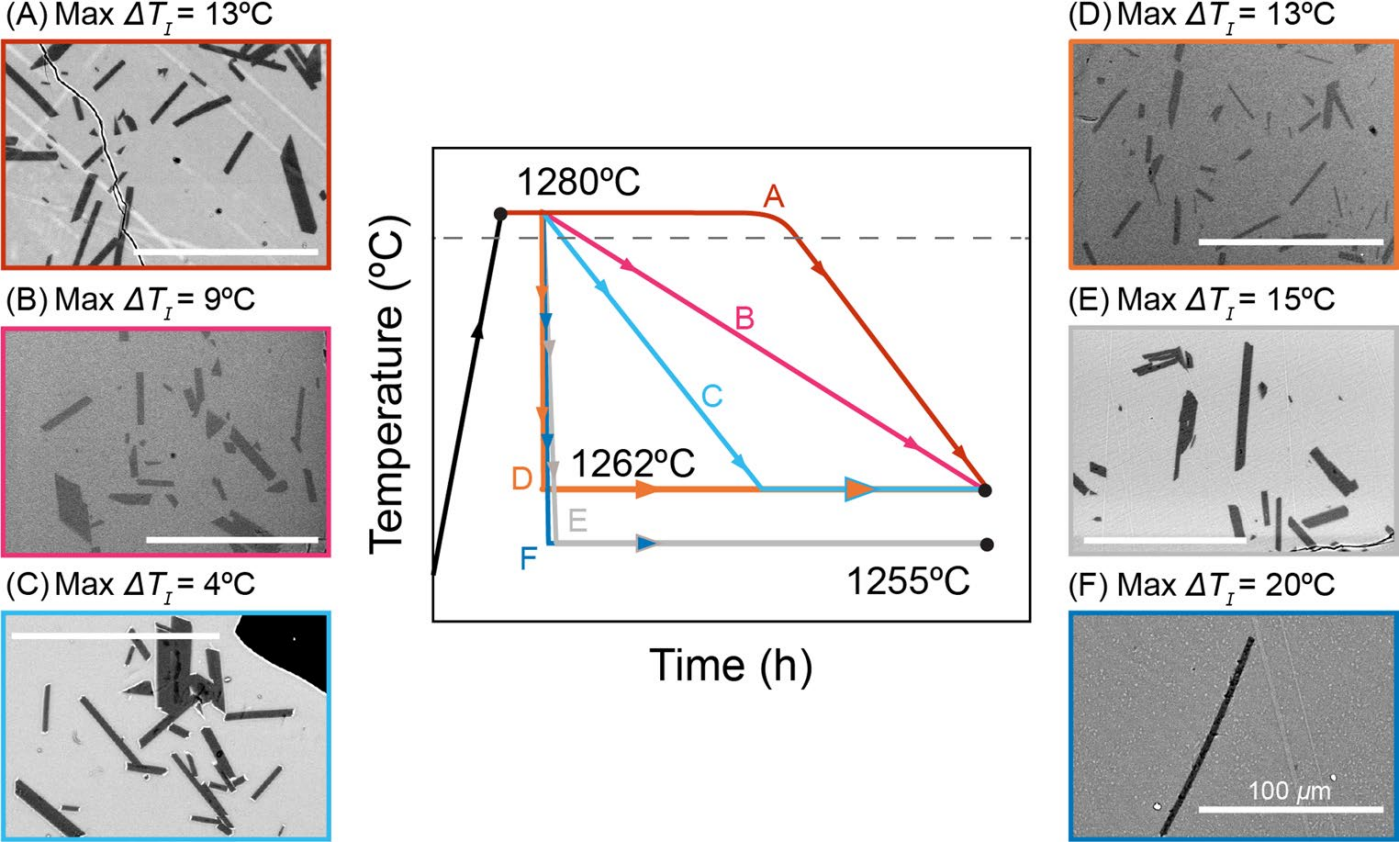
Schematic of 1-atm run pathways which involved continuous cooling ramps (blue, grey, light blue, pink, red) and single-step (orange) methods. The grey dashed line marks the liquidus temperature of the BEAT1-2 composition, where plagioclase is the first phase to nucleate. Representative plagioclase textures from experiments (**A** 1atm-3, **B** 1atm-1, **C** 1atm-2, **D** 1-atm-4, **E** 1atm-9, and **F** 1atm-8) are shown along with their maximum $\Delta T_{I}$ calculated from the crystallization model

**Fig. 6:**
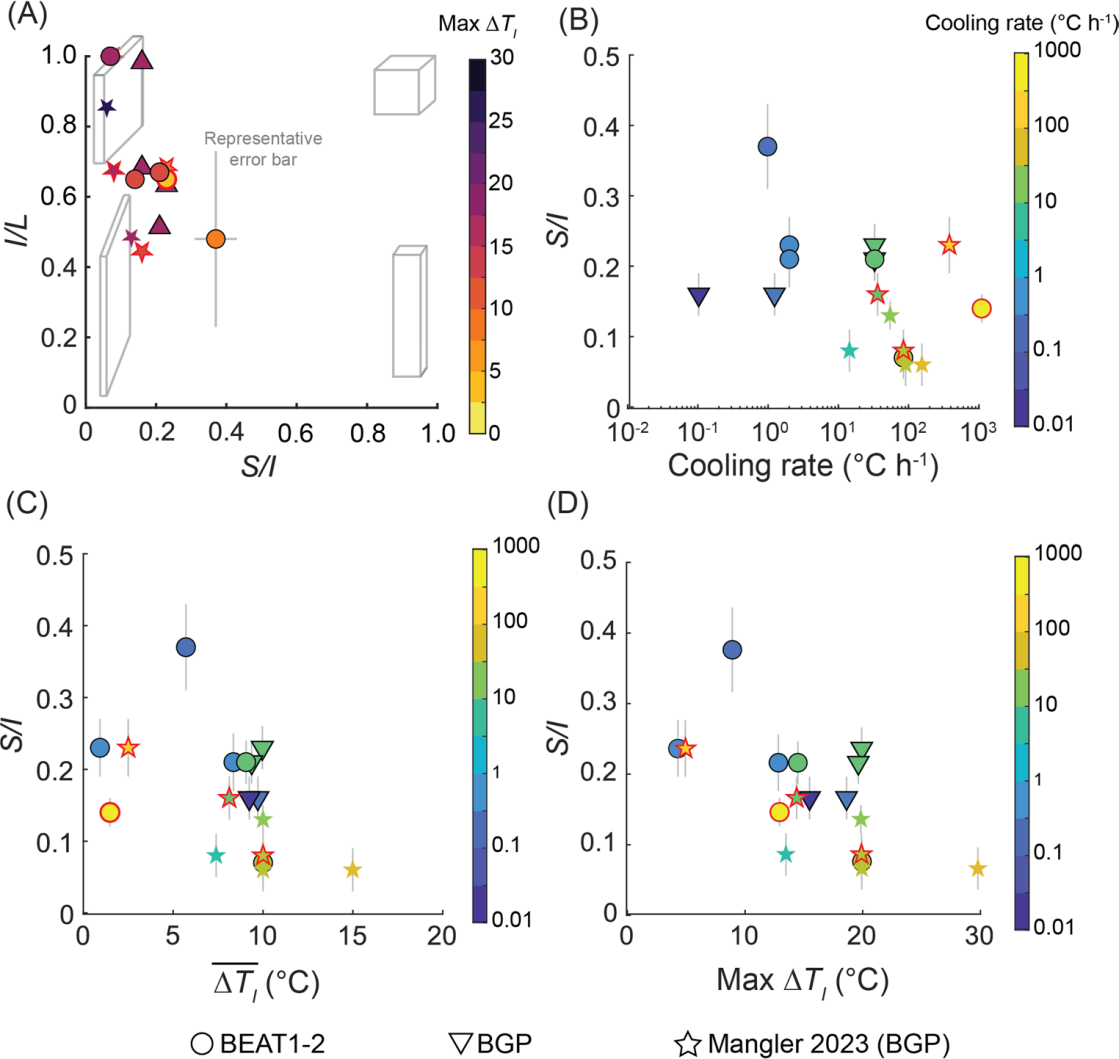
**A** Zingg diagram plotting short/intermediate (*S/I*) versus intermediate/long (*I/L*) crystal dimension ratios, allowing classification of 3D shape. Crystal shapes are classified as equant, tabular, bladed, or prismatic based on their position within the diagram (grey cuboids). **B** Crystal shape (*S/I*) as a function of cooling rate. **C** Crystal shape as a function of time-averaged undercooling ($\overline{{\Delta T_{I} }}$), where low $\overline{{\Delta T_{I} }}$ produces higher *S/I*; equant shapes (lower aspect ratios), whereas higher $\overline{{\Delta T_{I} }}$ results in more tabular, lower *S/I* value (higher aspect ratio) crystals. **D** The maximum $\Delta T_{I}$ that occurred during the simulation as a function of shape. The maximum $\Delta T_{I}$ and $\overline{{\Delta T_{I} }}$ are better indicators of crystal shape than cooling rate. Symbols with red borders indicate experiments that had higher crystal fractions than predicted by MELTS

**Fig. 7:**
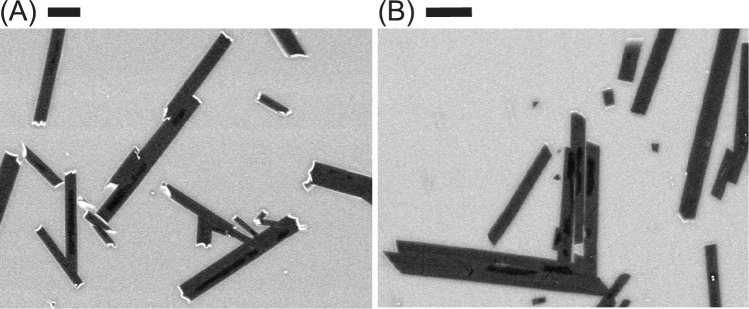
Backscattered electron (BSE) images of plagioclase crystals with dark, low anorthite content cores (**A** 1atm-2; **B** 1atm-9) Scale bars represent $10 \mu m$

**Fig. 8:**
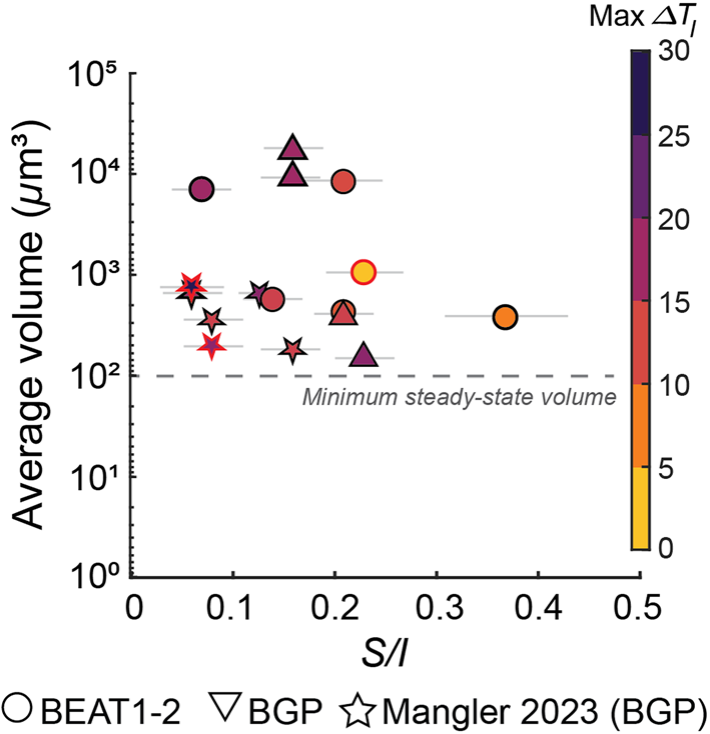
Crystal shape (*S/I*) as a function of average crystal volume. The grey dashed line delineates the minimum volume $\left( {100 \mu m^{3} } \right)$ expected a steady-state shape (Mangler et al. 2023). Our data show no systematic trend towards lower *S/I* values with average volume, consistent with mature shapes across runs. Symbol colors indicate the maximum $\Delta T_{I}$ calculated from the crystallization model

**Fig. 9:**
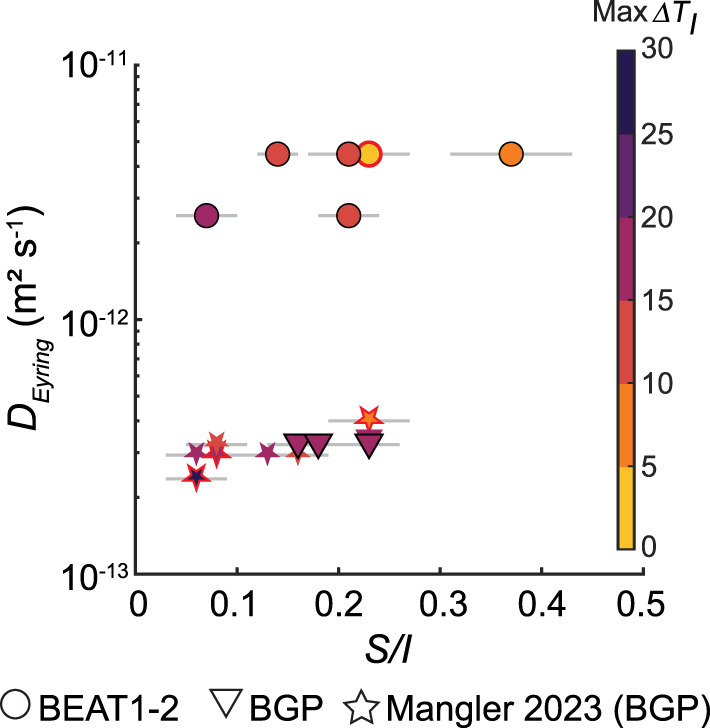
Crystal shape (*S/I*) as a function of melt diffusivity ($D_{Eyring}$). Symbols are colored by their calculated maximum $\Delta T_{I}$ value. Red borders denote experiments that had higher crystal fractions than the *MELTS* predicted value. Crystal shapes are similar despite a range of melt diffusivities ($10^{ - 13} - 10^{ - 11}$ m^2^ s^−1^)

**Fig. 10:**
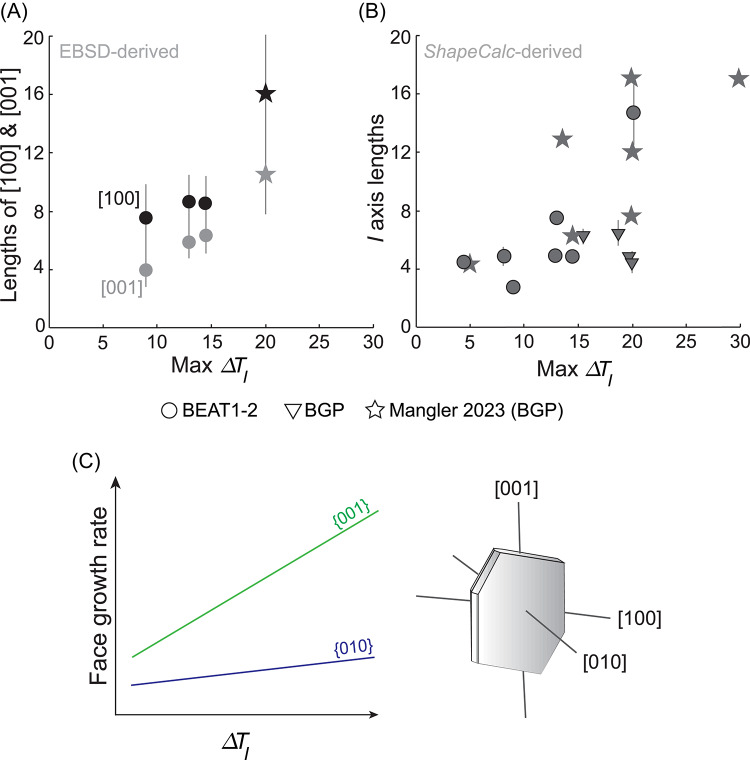
**A** EBSD-derived lengths along [100] and [001] crystallographic axes, normalized to [010], plotted against the maximum instantaneous undercooling ($\Delta T_{I}$). Lengths increase relative to [010], which we propose is due to an increase in the prevalence of the birth-and-spread growth mechanism. **B** The same trend is evident in the *ShapeCalc*-derived 3D intermediate (I) shape dimension, which is normalised to the shortest dimension (S). **C** Conceptual illustration of growth rates for plagioclase crystal faces {010} (blue) and {001} (green) as a function of $\Delta T_{I}$, redrafted from Higgins and Chandrasekharam (2007)

Our experiments consistently produced polyhedral plagioclase crystals, with no evidence of diffusion-limited growth (Fig. 5, see Online Resource 1 and 2 for representative BSE images), and minor amounts (< 1%) of iron-oxides. Cooling experiments utilizing the BEAT1-2 composition produced crystal shapes with *S/I* ratios ranging from 0.07 (±0.03) to 0.37 (±0.06), and *I/L* ratios from 0.48 (± 0.25) to 1.00 (± 0.20) (Fig. 6A; Table 4; see Online Resource 2 for length/width data). Crystals formed from the BGP composition exhibited more constrained shapes, with *S/I* ratios between 0.15 and 0.23 (± 0.03). EBSD analysis indicated that the longest dimension parallel to a crystallographic axis for plagioclase was [100], followed by [001], with the shortest being [010].

We observed a relationship between crystal shape and the maximum and averaged instantaneous undercooling (Fig. 6C and 6D). As expected, higher $\overline{{\Delta T_{I} }}$ values are typically generated by faster cooling rates and produced more tabular crystals with lower *S/I* values. Conversely, samples with lower $\overline{{\Delta T_{I} }}$, corresponding to slower cooling rates, yielded more equant shapes (higher *S/I*). However, when plotting *S/I* against cooling rate, it is evident that cooling rate is a less precise predictor of crystal shape compared to the max or $\overline{{\Delta T_{I} }}$ (Fig. 6). While $\overline{{\Delta T_{I} }}$ robustly characterizes most experimental cooling histories, experiments involving distinct cooling paths – such as the single-step method (e.g., 1atm-4) – are better described using the maximum $\Delta T_{I}$. We expect that recasting this relationship with the crystallinity-based supersaturation term of Befus and Andrews (2018) would yield a similar relationship between $\Delta \varphi$ and shape.

The average inferred volume of crystals produced in our cooling experiments was smaller than $V \approx { }1,000 \mu {\text{m}}^{3}$(Table 4). Larger crystals exceeding this volume formed only under conditions of very slow cooling rates (BGP-29; $V \approx 17,000 \mu {\text{m}}^{3}$; $\Delta T\Delta t^{ - 1}$ $= 0.09^\circ {\text{C h}}^{ - 1}$) or during extended hold periods following cooling ramps (e.g. experiment 1atm-3 $; V \approx 8,400 \mu m^{3}$;$12 {\text{h}}$ hold). Comparable volumes were reported by Mangler et al. (2023).

Equilibrium plagioclase volume fractions at the end of each experiment were estimated using phase equilibria generated from *MELTS* ($\phi_{Eq} ,$ Table 4). Most experiments exhibited low crystal fractions ($\phi < 0.1$). In BEAT1-2 experiments, crystallinities mostly fell below equilibrium predictions, which may reflect disequilibrium crystallization and is reflected in our simulations. Experiments using the BGP composition consistently resulted in crystallinities below predicted equilibrium values (Table 3).

**Table 3:** Comparison of *ShapeCalc* and EBSD-derived shape parameters

	*ShapeCalc*								EBSD						
Run no.	*S*	*I*	*L*	*S/I*	SD^*SI*^	*I/L*	SD^*IL*^	*R*_*c*_^*2*^	*b*	*c*	*a*	*S/I*	SD^*SI*^	*I/L*	SD^*IL*^
1atm_1	1	2.7	5.6	0.37	0.06	0.48	0.25	0.978	1	3.9	7.4	0.23	0.04	0.74	0.26
1atm_2	1	4.4	6.8	0.23	0.04	0.65	0.21	0.974	1	5.7	8.5	0.18	0.04	0.64	0.14
1atm_3	1	4.8	7.2	0.21	0.04	0.67	0.2	0.909	1	9.1	18.5	0.08	0.04	0.79	0.20
1atm_4	1	7.4	16	0.14	0.02	0.65	0.23	0.989	–	–	–	–	–	–	–
1atm_8	1	14.5	15	0.07	0.03	0.97	0.2	0.924	–	–	–	–	–	–	–
1atm_9	1	4.8	7.2	0.21	0.03	0.67	0.21	0.977	1	6.3	8.4	0.20	0.04	0.70	0.20
1atm_BGP26	1	4.4	6.8	0.23	0.03	0.65	0.21	0.935	–	–	–	–	–	–	–
1atm_BGP27	1	6.4	6.4	0.16	0.03	1.00	0.19	0.874	–	–	–	–	–	–	–
1atm_BGP28	1	4.7	8.8	0.21	0.03	0.53	0.19	0.896	–	–	–	–	–	–	–
1atm_BGP29 < 8.7 µm	1	8.4	8.4	0.12	0.03	1.00	0.22	0.960	–	–	–	–	–	–	–
1atm_BGP29 > 8.7 µm	1	5.0	6.4	0.20	0.03	0.78	0.19	0.938	–	–	–	–	–	–	–
BGP3^§^	1	17	20	0.06	0.03	0.85	0.20	0.941	1	10.4	15.9	0.09	0.02	0.65	0.19

^§^Sample from Mangler et al. (2023)

Experiments 1atm-2 and 1atm-9 contained crystal cores with lower anorthite content, identified based on their darker grayscale values in BSE images (Fig. 7). We interpret these cores as relic crystals that formed during the initial heating and persisted through the subsequent supraliquidus step. Crystals grown during heating experience high $\Delta T_{I}$, commonly resulting in anomalous compositions (Smith and Brown 1988). Thus, these cores represent early-stage growth under conditions of elevated undercooling. These cores had an average volume of approximately $15 \mu {\text{m}}^{3}$, sufficiently small that they are unlikely to have significantly affected overall crystal shape. Indeed, the crystal shape determined for experiment 1atm-2 was statistically indistinguishable from a nearly identical apparently core-free experiment (1atm-3). Consequently, we conclude the presence of small cores in our experiments did not substantially influence the measured crystal shapes.

## Discussion

Our results demonstrate clear relationships between the maximum and time-averaged instantaneous undercooling and crystal shape (Fig. 6C and 6D). Several factors could explain these observed trends: (1) progressive shape evolution during growth: Mangler et al. (2022) demonstrated that plagioclase crystal shapes transition from initially prismatic forms following nucleation to more tabular, steady-state shapes (expressed as *S/I* ratios) with continued growth. The extent of shape evolution is influenced by nucleation density, where lower nucleation densities allow greater shape changes through increased available growth volume per crystal (Mangler et al. 2022). We assess how variations in nucleation density may have influenced crystal shapes in our experiments. (2) Impact of melt diffusivity on crystal morphology: Mangler et al. (2023) showed that steady-state crystal shapes, which depend on the relative growth rates along the *S* and *I* dimensions, may be sensitive to melt composition or melt diffusivity. Melt compositions remained relatively consistent across our experiments and compiled literature datasets, however, temperature variations could have impacted melt diffusivity. We evaluate whether these diffusivity differences could explain the shape variations observed. (3) Influence of instantaneous undercooling on crystal growth mechanisms: instantaneous undercooling directly influences the growth mechanisms operating on crystal faces, affecting the faces’ relative growth rates and therefore overall crystal shape. We explore how shifts in growth mechanisms at varying $\Delta T_{I}$ contribute to overall shape trends. In the following sections, we utilize our compiled dataset and evaluate these hypotheses in detail to clarify their contributions to the observed relationships between crystal shape and $\overline{{\Delta T_{I} }}$.

### Incomplete shape evolution at crystal size $<\,100 \,\mu m^{3}$

As outlined above, crystals in samples with low crystal number density quickly achieve their tabular, steady-state shape, even at small crystallization increments, whereas higher number densities at similar crystallinity ($\phi$), can delay shape maturation by limiting the available growth volume per crystal. Crystals below the minimum volume (~100 µm^3^) for steady-state shape typically show a negative correlation between *S/I* and volume and a spread in *S/I* values (Fig. 8; Mangler et al. 2023). In our experiments, crystals exceed 100 *µ*m^3^ and we observe essentially no relationship between *S/I* and average volume between experiments, indicating that the measured shapes are mature rather than early, pre-steady-state forms. We therefore attribute differences among experiments to be driven by other factors, not to incomplete shape evolution.

### Does melt diffusion moderate crystal morphology?

Mangler et al. (2023) demonstrated that when melt diffusivity is similar to interfacial reaction rates, relative differences in interfacial energies between plagioclase crystal faces can result in slower-growing interfaces to remain interface-controlled, whereas faster-growing faces may shift to diffusion-limited growth. Our study exclusively investigated basaltic melts (cf. basalt and dacite studied by Mangler et al. 2023); therefore, we anticipate any differences in plagioclase interfacial energies arising from compositional differences to be trivial. However, since the experiments span a range of temperatures, it is important to evaluate whether temperature-dependent variations in melt diffusivity might have influenced crystal shapes.

To test this, we calculated melt diffusivities ($D_{Eyring}$) for each experiment (Table 4) using equation 5, with melt viscosities derived from the Giordano et al. (2008) model. We found no clear relationship between $D_{Eyring}$ and resulting crystal shapes within our dataset or Mangler et al. (2023; Fig. 9). Overall, our analysis indicates that melt diffusivity alone does not control the crystal shapes observed across the compositional and temperature ranges explored in this study.

**Table 4:** Textural analyses of plagioclase crystals

Run no.	$D_{Eyring}$ (m^2^ s^−1^)	Analysed area (mm^2^)	*n*	Crystal area (mm^2^)	$S_{n}$* (µm)	$\phi^{{\text{\S}}}$	$\phi_{Eq}$	$N_{A}$ (mm^−2^)	$N_{V}$ (mm^−3^)	Average volume (µm^3^)
1atm_1	4.5e−12	0.58	470	0.0238	7.1	0.041	0.060	810	1.14e5	360
1atm_2	4.5e−12	1.11	692	0.0656	10.3	0.066	0.060	624	6.41e4	1023
1atm_3	4.5e−12	1.60	181	0.0736	20.4	0.047	0.060	113	5.63e3	8385
1atm_8	2.6e−12	7.07	7	0.0002	33.9	0.001	0.092	1	1.67e2	210
1atm_9	2.6e−12	0.70	958	0.0505	7.3	0.072	0.092	1369	1.89e5	382
1atm_BGP26	3.2e−13	0.13	12	0.0003	5.2	0.003	0.085	93	1.77e4	144
1atm_BGP27	3.2e−13	1.22	43	0.0168	21.3	0.016	0.085	35	1.78e3	8973
1atm_BGP28	3.2e−13	0.16	106	0.0050	7.4	0.037	0.085	667	9.73e4	380
1atm_BGP29	3.2e−13	2.71	161	0.1066	25.7	0.039	0.085	59	2.31e3	17041
1atm_4	4.5e−12	0.23	240	0.0109	9.0	0.086	0.060	1050	1.56e5	552

*$S_{n}$; average crystal diameter

^§^$$\phi$$; crystallinity

### Influence of instantaneous undercooling on growth

Simultaneously measuring growth rates on adjacent crystal faces remains challenging in experimental studies due to the limitations in capturing evolving 3D crystal shapes (Kirkpatrick 1976; Watanabe and Kitamura 1992; Park and Hanson 1999; Cabane et al. 2005; Schiavi et al. 2009). Identifying dominant growth mechanisms typically requires systematic tracking of crystal sizes (e.g., radii) over the course of crystallization experiments. While extensive time-series experiments were beyond the scope of our study, we inferred changes in the growth rates or mechanism by comparing relative crystal lengths along the [001] and [100] crystallographic axes relative to the shortest axis ([010]) (Fig. 4).

At low to moderate maximum instantaneous undercooling, EBSD measurements indicate a gradual increase in crystal elongation along [001] relative to [010](Fig. 10A), resulting in progressively more tabular shapes—a trend similarly observed in *ShapeCalc*-derived data (Fig. 10B). We interpret this increase in anisotropy as reflecting a greater prevalence of birth-and-spread growth, and increase in relative growth rates, on faces other than {010}.

Our data support the conceptual framework proposed by Higgins and Chandrasekharam (2007), wherein growth rates along primary crystallographic axes [001] and [010] vary systematically with increasing $\Delta T_{I}$ (Fig. 10C). As $\Delta T_{I}$ increases, birth-and-spread growth increasingly dominates along [100] and [001], whereas growth along [010] remains predominantly controlled by screw dislocations, even at relatively high $\Delta T_{I}$ (Fig. 10). Our results demonstrate how increased $\Delta T_{I}$ enhances crystal elongation, suggesting shifts in the prevalence of different growth mechanisms along different crystallographic directions primarily control the relationship between the max $\Delta T_{I}$/$\overline{{\Delta T_{I} }}$ and resultant polyhedral crystal shape.

### Application to natural systems

**Fig. 11:**
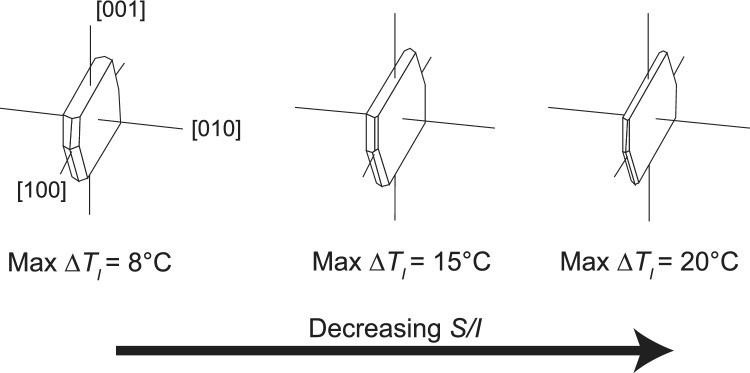
Idealised evolution of plagioclase shape as a function of maximum instantaneous undercooling (Max $\Delta T_{I}$), based on the relative lengths along crystallographic axes [100],[010], and [001] determined from EBSD measurements. With increasing max $\Delta T_{I}$, crystals become progressively more elongated (lower *S/I* values)

For the first time, we establish a quantitative relationship between instantaneous undercooling and crystal shape within cooling rate ranges representative of naturally solidifying magmas in the Earth’s crust. In slowly cooled magmatic intrusions, crystallization predominantly occurs within an interface-controlled growth regime. Under such conditions, low instantaneous undercooling dictates the prevalence of different growth mechanisms operating on each crystal face, thus influencing their relative growth rates and ultimately controlling crystal shape. Specifically, lower $\overline{{\Delta T_{I} }}$ (achieved through slower cooling rates and thus lower $\Delta T_{I}$) produce more equant crystals with higher *S/I* values (or lower aspect ratios), due to less anisotropic growth. Consequently, longer crystallization timescales generally favour the formation of relatively more equant crystal shapes. At higher $\overline{{\Delta T_{I} }}$, growth is more anisotropic, perhaps because the faster birth-and-spread mechanism comes to dominate most faces, while growth along [010] remains slow (Fig. 11).

Cashman (1993) proposed the importance of $\Delta T_{I}$ in controlling plagioclase growth rates, laying critical foundation for quantitatively linking thermal history to crystal shape. Our results further reinforce the utility of plagioclase shape and aspect ratio as quantitative indicators of thermal evolution in magmatic systems. For example, Holness (2014) documented a linear relationship between plagioclase aspect ratio and the logarithm of crystallization time inferred from 1D cooling models of sills. Similarly, near-symmetrical "M-shape" profiles—where plagioclase aspect ratios vary systematically with intrusion depth—have been observed in other natural systems, including the Makaopuhi lava lake and basaltic samples recovered from deep-sea drilling (Kirkpatrick 1976; Coish and Taylor 1979; Cashman and Marsh 1988; Zieg and Marsh 2002; Holness 2014; 2017). Similar trends have also been documented in other silicate phases, such as amphibole and orthopyroxene (Zhang et al. 2019; Okumura et al. 2022).

Introducing $\overline{{\Delta T_{I} }}$ as a quantifiable metric provides a powerful new way to express cumulative driving forces for crystallization. Unlike relying solely on cooling rate, $\overline{{\Delta T_{I} }}$ allows crystallization conditions to be expressed directly in terms of undercooling, opening avenues for interpreting various magmatic processes including water-saturated decompression (Andrews and Befus 2020). When combined with other metrics such as size distribution and nucleation density, crystal shapes can quantitatively constrain cooling or ascent paths for natural magmas. For erupted magmas, these constraints could further be linked retrospectively with geophysical observations, providing insight into conduit dimensions and eruptive processes. Further refinement of the shape- $\overline{{\Delta T_{I} }}$ trend would benefit from future studies focusing on constraining parameters used for calculating nucleation and growth rates (Eqs. 2 and 3) as well as nucleation delay (Eq. 4).

Finally, we emphasise that the underlying mechanisms identified here controlling polyhedral plagioclase shapes at low undercooling are expected to apply broadly to other anisotropic minerals. Similar approaches could be extended to other common silicates such as clinopyroxene, olivine or zircon, expanding interpretations of crystallization histories in diverse geological settings. Future studies should also consider complexities such as core-overgrowth and surface roughness influence on growth mechanisms, changes in melt diffusivity during crystallization, and crystal impingement at high crystallinity. Furthermore, developing efficient methods to identify specific crystal faces directly from 2D EBSD maps would advance our understanding of how instantaneous undercooling influences the growth rate of individual crystal faces. Additionally, incorporating anisotropic growth into our framework would enable coupled evolution of crystal size distributions and shape as a function of the undercooling history ($\overline{{\Delta T_{I} }}$). Such modelling is critical for reconstructing magma ascent histories in conduit models.

## Conclusion

Our study establishes average instantaneous undercooling ($\overline{{\Delta T_{I} }}$) as a quantitative measure of the thermodynamic driving force controlling polyhedral plagioclase shape, providing a transformative framework for interpreting crystallization processes in magmatic systems. Through detailed analysis of growth along different crystallographic directions, we demonstrate that higher $\overline{{\Delta T_{I} }}$ increases contrasts in face-specific growth rates, producing tabular crystals (lower *S/I* or higher aspect ratio). Conversely, lower $\overline{{\Delta T_{I} }}$ promotes relatively more isotropic growth across faces and produces more equant shapes. Our results experimentally validate a causal link between crystallization timescales and plagioclase shape, confirming that crystal shape serves as a quantifiable proxy for the cooling histories of a magmatic system.

Beyond plagioclase, similar shifts in growth mechanisms likely influence crystal shapes in other anisotropic silicate minerals. $\overline{{\Delta T_{I} }}$ therefore has the potential to serve as a universal framework for interpreting crystal textures across diverse magmatic environments. Moreover, because $\overline{{\Delta T_{I} }}$ captures cumulative crystallization explicitly in terms of undercooling rather than cooling rate alone, it provides a powerful tool for reconstructing thermal histories. Although demonstrated here under anhydrous, ambient pressure conditions, $\Delta T_{I}$–defined relative to the saturation conditions of the crystallizing mineral under the prevailing pressure, melt composition, and crystallinity - is readily extendable to ascent and degassing scenarios.

## Supplementary Information

Below is the link to the electronic supplementary material.

Supplementary file1 (M 3 kb)

Supplementary file2 (M 11 kb)

Supplementary file3 (XLSX 12 kb)

Supplementary file4 (DOCX 453 kb)

Supplementary file5 (XLSX 7379 kb)

Supplementary file6 (TXT 1 kb)

## Data Availability

Data and code used for the production of this manuscript have been made available in the supplementary documentation.
