# Supplementary material for: Linking crystal shape and dynamic undercooling: a new framework for inferring magmatic crystallization histories
Source: Contrib Mineral Petrol. 2025 Dec 2;180(12):92. doi: 10.1007/s00410-025-02278-6 (PMC12669325; doi:10.1007/s00410-025-02278-6)
Supplement: Supplementary file 4 — Supplementary file4 (DOCX 453 kb) [file 410_2025_2278_MOESM4_ESM.docx]

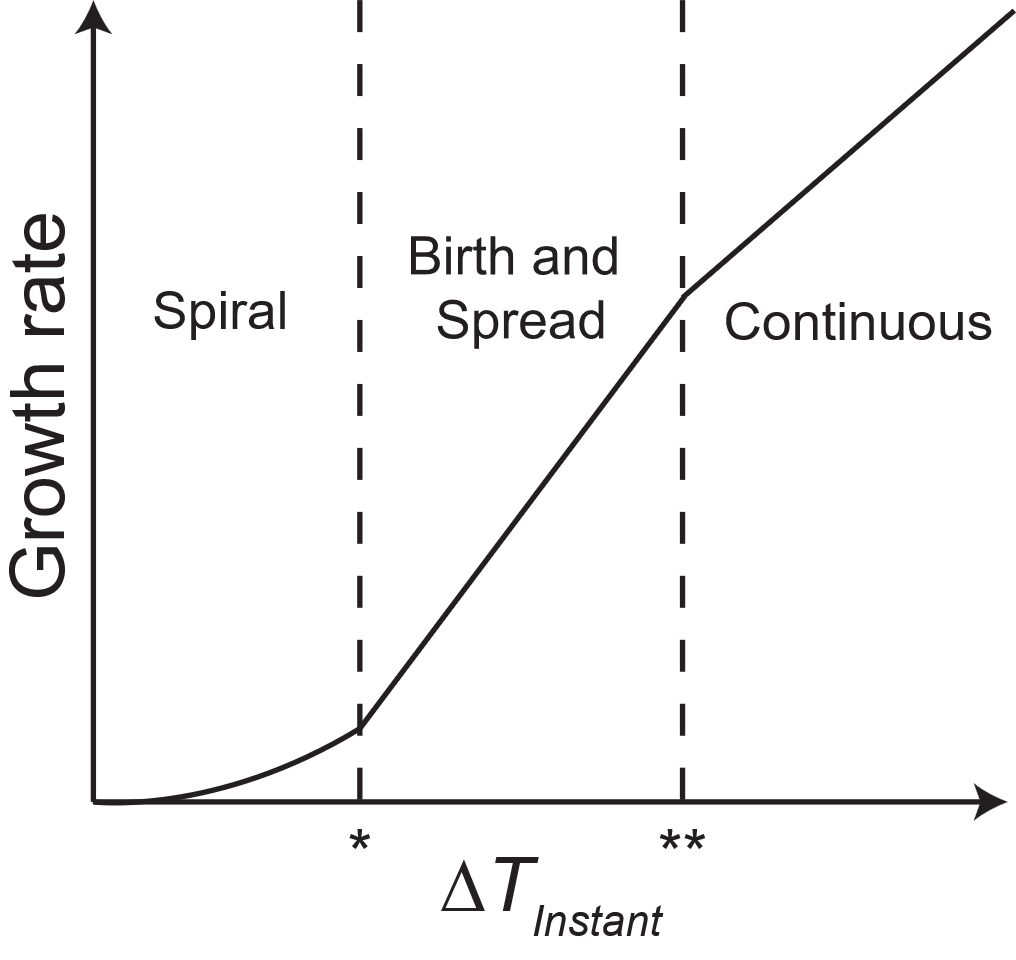


**Supplementary Figure 1** Growth rate vs instantaneous undercooling (${\Delta T}_{I}$), redrawn from Sunagawa (2009, Fig.3.15). Three distinct growth mechanisms are highlighted: spiral (screw dislocation), birth-and-spread (2D surface nucleation), and continuous (rough) growth. Asterisks (*, **) denote the thresholds at which growth mechanisms transition, depending on the degree of ${\Delta T}_{I}$


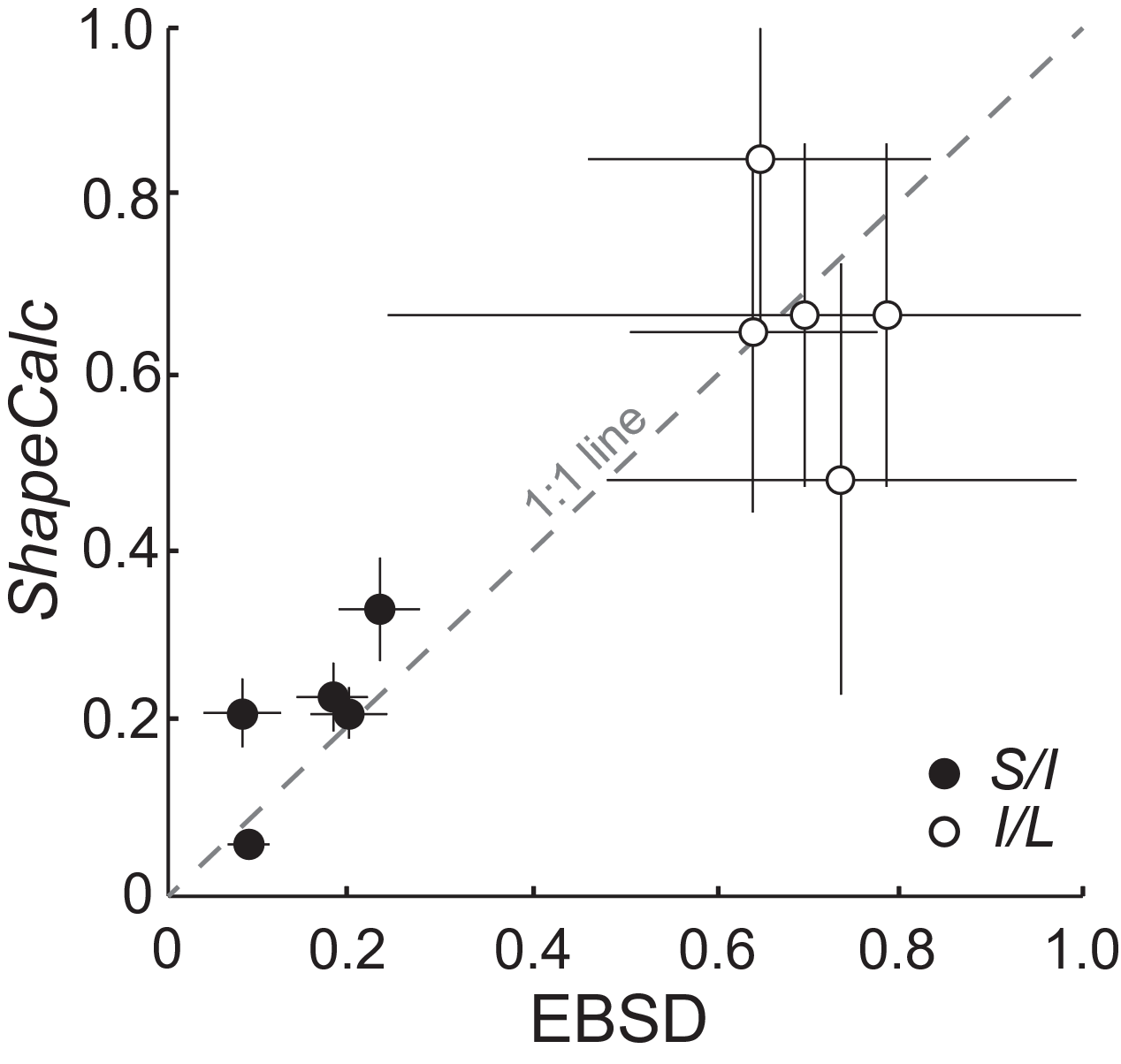


**Supplementary Figure 2** Comparison of short/intermediate S/I (closed symbols) and intermediate/long I/L (open symbols) dimension ratios obtained from ShapeCalc and EBSD measurements. I/L uncertainties are large regardless of method, reflecting the inherently lower likelihood of intersecting the longest crystal dimension from random 2D sections

**
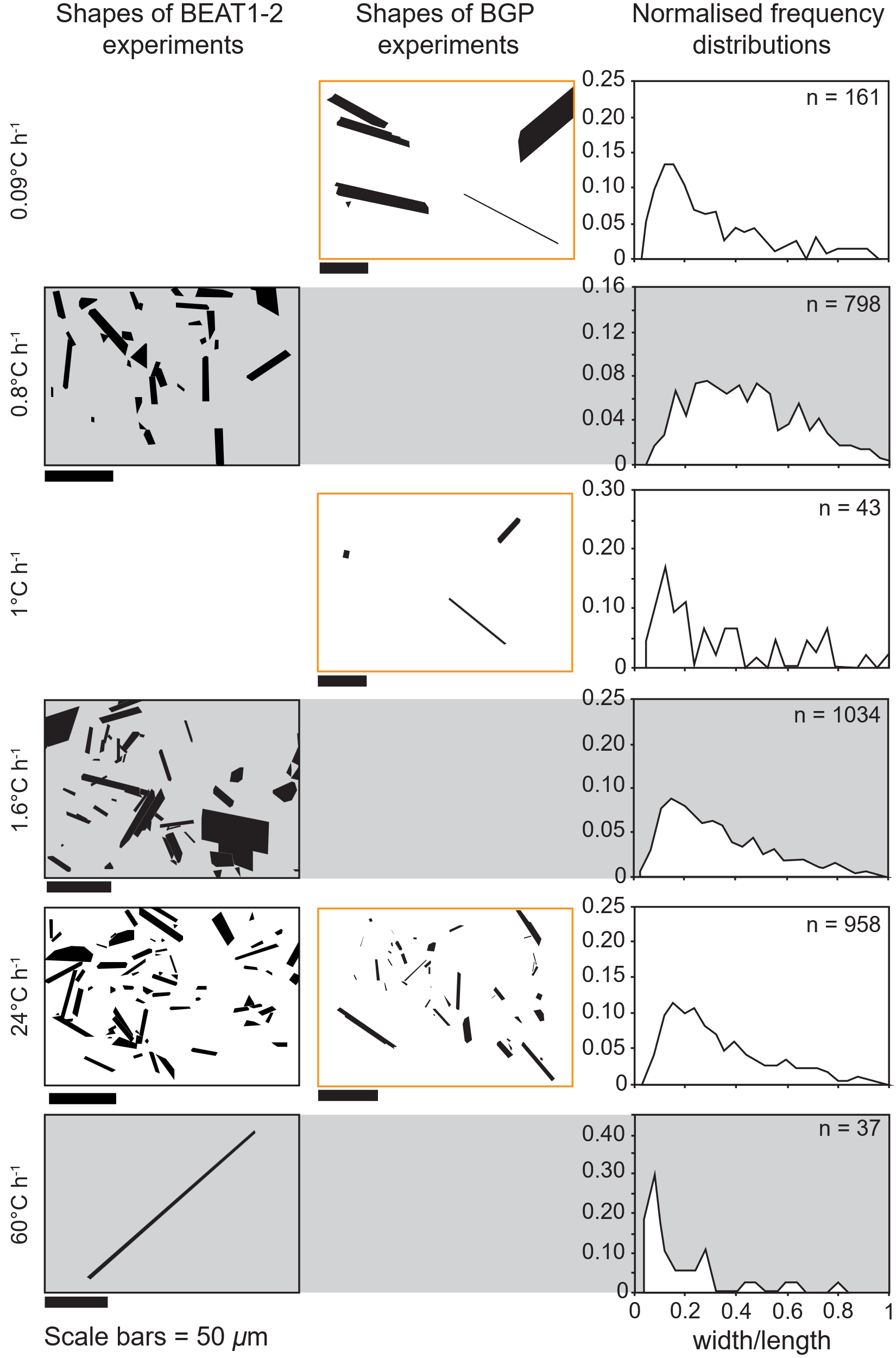
**

**Supplementary Figure 3** Binary images showing representative plagioclase textures from experiments conducted at a range of cooling rates ($0.09 - 60 ^{\circ}C h^{-1}$). Black borders indicate experiments using the BEAT1-2 composition (in descending order:1atm-1, 1atm-2, 1atm-9, and 1atm-8), whereas orange borders represent those using the BGP composition (BGP-29, BGP-27, BGP-28). Histograms show the samples’ corresponding normalized frequency distribution of width/length ratios derived from BSE images
